# Computational Approaches to the Electronic Properties of Noble Metal Nanoclusters Protected by Organic Ligands

**DOI:** 10.3390/nano11092409

**Published:** 2021-09-16

**Authors:** Francesco MUNIZ-MIRANDA

**Affiliations:** Department of Applied Science and Technology, Politecnico di Torino (PoliTO), Corso Duca degli Abruzzi 24, 10129 Turin, Italy; francesco.munizmiranda@polito.it

**Keywords:** nanoclusters, metal, organic ligands, optical properties, TD-DFT

## Abstract

Organometallic nanoparticles composed by metal cores with sizes under two nanometers covered with organic capping ligands exhibit intermediate properties between those of atoms and molecules on one side, and those of larger metal nanoparticles on the other. In fact, these particles do not show a peculiar metallic behavior, characterized by plasmon resonances, but instead they have nonvanishing band-gaps, more along molecular optical properties. As a consequence, they are suitable to be described and investigated by computational approaches such as those used in quantum chemistry, for instance those based on the time-dependent density functional theory (TD-DFT). Here, I present a short review of the research performed from 2014 onward at the University of Modena and Reggio Emilia (Italy) on the TD-DFT interpretation of the electronic spectra of different organic-protected gold and/or silver nanoclusters.

## 1. Introduction

**Figure 1:**
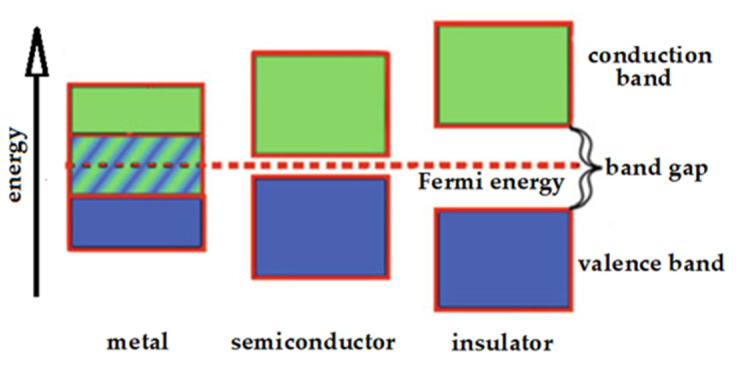
Band-gaps in metals, semiconductors and insulators.

Metal clusters with small sizes, indicatively with diameter smaller than two nanometers, show a peculiar behavior that is different from that of what are commonly called nanoparticles. In fact, they exhibit negligible plasmon resonances, because their sizes are comparable to the Fermi wavelength of electron (ca. 0.7 nm). On the contrary, they often present nonzero band-gaps more like semiconductors, as well as atoms and molecules. In fact, the band gap usually decreases when the particle size increases and vice versa. The typical behaviors of band-gaps in metals, semiconductors and insulators are depicted in Figure 1.

Metals and metal oxides are generally considered the best materials for the manufacture of catalysts for the most varied reactions. In particular, heterogeneous catalysis is exclusively based on the reactivity of surface atoms. For this reason, it is important to have a high degree of dispersion of the metal or a very small particle size. Metal nanoclusters meet these needs, because they belong to a nanometer range of approximately 1–5 nm [1]. Many factors have significant influences on the catalytic properties of these metal catalysts, particle size, shape, chemical composition, interaction of metal with reactant or dispersing medium.

Nanomaterials have electrical transport properties that can be exploited in the fabrication of single electron (SE) devices, for example single electron transistors, which retain their scalability down to the molecular level. Particular importance in this field are metal nanoclusters, which can be synthesized with dimensions of 2 nm or even less and which have a large charging energy as determined by the particle size. Hence, they fulfill the requisites as functional elements in nanoelectronics [2].

Metal nanoclusters exhibit strong quantum confinement effects [3], with optical, electronic, and chemical properties different from larger nanoscale metals. For example, metal NCs exhibit discrete energy levels of electrons and several molecular-like properties. Luminescence is an important property of metal nanoclusters and underlies many biomedical applications, such as in biomedical sensing, bioimaging, drug delivery and nanotherapy. In addition, they exhibit efficient luminescence emission from visible to near-infrared light [4].

Most metal clusters are covered with organic ligands such as phosphines and thiols, which maintain their size at the nanometer scale and at the same time prevent the formation of larger particles by aggregation. It is also important to pinpoint that the structures of these system are experimentally resolved: this means that their geometries are not the product of some toy-model, nor are they created with a molecular builder out of fantasy, but they are actual objects that can be synthesized and crystallized to allow X-ray scattering resolution.

In fact, the structures of these objects, as well as the possibility to predict them, has been largely rationalized within the so-called “superatom” concept, introduced by Hannu Hakkinen and collaborators in 2008 [5,6]. This theoretical framework considers the gold-core as a sort of giant atom which becomes particularly stabilized when its number of electrons and electron-withdrawing groups matches certain magic numbers. In particular, in its original formulation a number n* was computed as

n* = *N*_*Au*_ − *W* − *q* (1)

where *N_Au_* is the number of gold atoms (and, as a consequence, the number of unpaired electrons), *W* is the number of electron-withdrawing groups, and *q* is the charge of nanocluster. The superatom concept states that the nanocluster is stable when n* is equal to a number of a specific set of “magic” integers such as 2, 8, 18, 34, and so on. Thus, this theoretical framework basically borrows the idea that the stability of noble gas is achieved in the presence of a certain number of electrons, and extends it to noble-metal nanoclusters. This ingenious idea is mainly applicable to almost-spherical objects, while it is not very predictive for very oblate or prolate nanoclusters. Further, it deals with their stability, which is a ground-state property, and does not address excited-state features.

Recently, an interpretation that draws inspiration from the quark subatomic models has been proposed for gold nanoclusters [7].

Anyway, organometallic nanoparticles can also have typically molecular optical properties such as the emission of fluorescence [8,9], with potential applications in both optoelectronics and biomedicine [10,11,12,13], especially when the metal core is composed of gold and/or silver atoms. The organic ligands interacting with the metal cores may play an important role in determining not just the three-dimensional structures of metal nanoclusters but also their electronic behavior [14,15]. Consequently, investigating the effect of the organic ligands on the structural stabilization of the metal nanoclusters is a prerequisite to understand their optical properties. The developments in quantum chemistry now allow study of these systems and predicting or interpreting experimental data that would otherwise be difficult to rationalize and understand [16,17].

Calculations based on density functional theory (DFT) probably represent the best compromise between accuracy and computational feasibility and, furthermore, the time-dependent extension (TD) to DFT calculations allows you to investigate excited electronic states to predict and analyze optical spectra of these systems [18,19,20]. This short review, based on the investigations performed at the University of Modena and Reggio Emilia, illustrates the application of the TD-DFT method to the study of the spectroscopic properties of ligand-protected gold and/or silver nanoclusters, both in absorption (extinction spectra) and in emission (fluorescence spectra).

## 2. Computational Details

Gaussian 09 software [21], within the DFT and TD-DFT frameworks, was employed for the calculations of the structural and spectroscopic properties of the ligand-capped nanoclusters. Geometry optimizations carried out on crystallographic structures have been performed with different exchange-correlation functionals [22], at both GGA (generalized gradient approximations) and hybrid level of theory. CAM-B3LYP range-separated hybrid functional was instead used for most of the TD-DFT spectroscopic calculations as it best captures possible charge-transfer phenomena [23], along with a number of localized basis sets (based on Gaussian functions).

## 3. Results

### 3.1. Absorption Spectra

**Figure 2:**
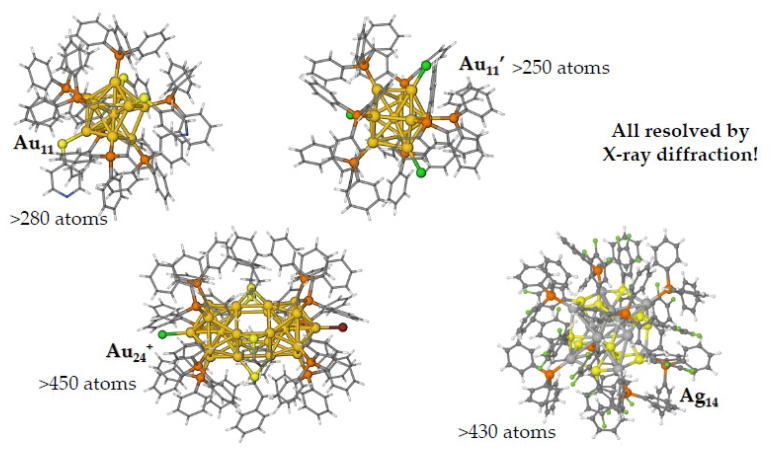
Different organometallic nanoparticles formed by noble metal cores protected by phenylphosphines.

Different nanoparticles formed by cores of Au or Ag atoms connected to phenylphosphines (Figure 2) have been studied by DFT calculations in order to reproduce both structural and optical properties [22].

**Figure 3:**
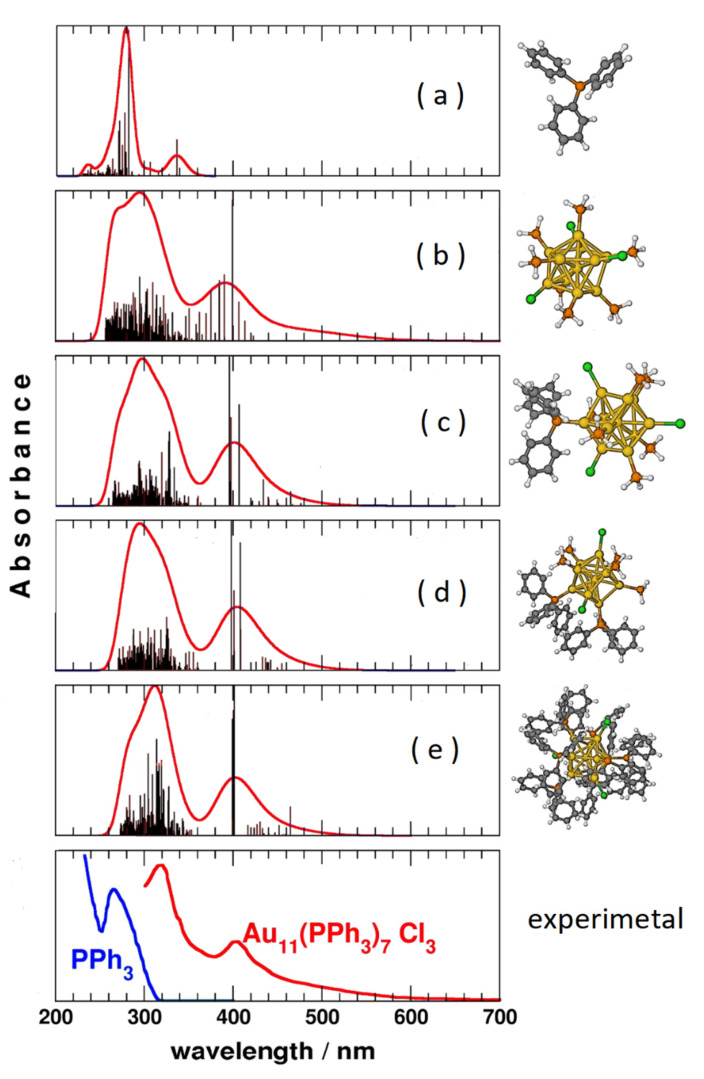
TD-DFT simulated absorption spectra of different model systems of the Au_11_(PPh_3_)_7_Cl_3_ nanoparticle, compared with the experimental ones. Spectra (**a**–**e**) are computed with the same level of theory on different models of triphenylphosphine and of the gold nanocluster.

In particular, accurate calculations of the excited electronic states were also performed on ligand-protected Au_11_ nanoclusters to try to understand whether the observed absorption bands are due to charge transfer or stacking interactions between ligand and metal. It has been shown that the transitions are mainly due to localized states on the gold core, while the ligands have very secondary effects [24]. This is illustrated in Figure 3: the TD-DFT spectra of the PPh_3_ ligands (panel a) are very different from those of the core Au_11_ (panel b), while all these models (panels c, d, e) are very similar to each other. The simplest model (panel b), without the organic part of the ligands, is already by itself able to reproduce the experimental spectrum (panel f) with acceptable accuracy, demonstrating that the role played by organic ligands can be considered negligible.

These conclusions cannot be generalized to all ligand-functionalized gold nanoclusters, because a case-by-case investigation remains necessary; still, these suggest that often gold nanocores give the largest contribution to low-energy excited states. It should be noted that nanoparticles with silver cores show that organic ligands have a much more relevant effect in electronic spectra, both in absorption and in emission.

**Figure 4:**
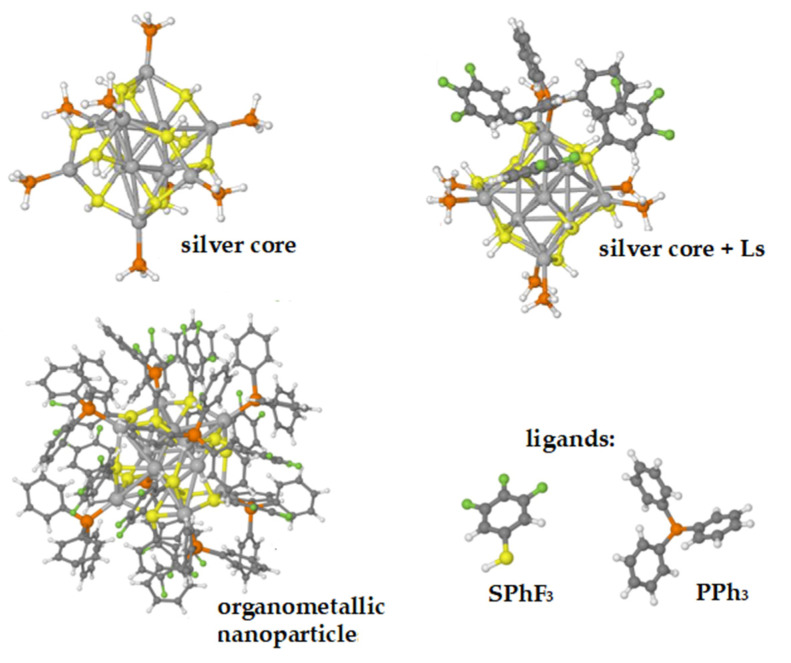
Three-dimensional structures of the Ag_14_(SPhF_2_)_6_ (SPhF_3_)_6_ (PPh_3_)_8_ nanoparticle and its components.

**Figure 5:**
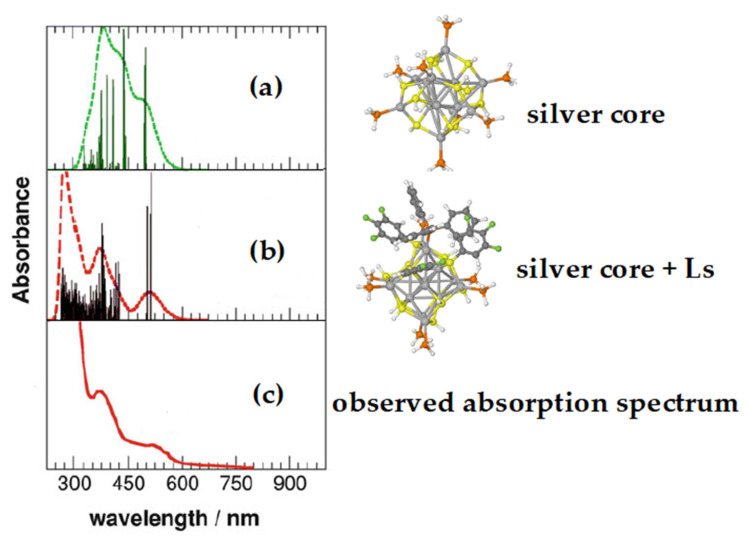
TD-DFT simulated absorption spectra of different model systems of the ligand-protected Ag_14_ nanocluster, compared with the experimental ones. Spectra (**a**,**b**) are computed on different models of the silver nanocluster, whereas the (**c**) spectrum is the experimental one taken from ref [25].

An example of this latter behavior is a nanoparticle formed by an Ag_14_ core connected to both fluorinated thiophenol and triphenylphosphine (see Figure 4), whose optical properties are interesting due to its luminescence [9]. In this case, as shown in Figure 5, the profile of the experimental UV-vis absorption spectrum can be well reproduced but only if in the computational model some ligands (Ls) are retained [25], as shown in panel (b).

As shown in panel a, the simulated spectrum that omits the ligands markedly differs from the experimental one. Actually, it was shown that silver and gold nanoparticles could have quite different optical behaviors: when the gold core is present, low fluorescence emission is usually observed, while silver nanoparticles are often very luminescent [9,26,27,28]. This different behavior may hinder the use of gold in nanoparticles used in application fields such as phototherapy, in spite of their better biocompatibility.

### 3.2. Fluorescence Spectra

By combining gold and silver atoms into a single metal core, it is possible to obtain an object that holds the best of both categories together. Wang et al. [29] produced two different mixtures of Ag_n_Au_25-n_^2+^ nanoclusters (“*nanoalloys*”) connected to different ligands (aromatic thiols and phosphines) with different synthesis methods. One product (product II) had a higher silver content than the other (product I), with a significant amount of Ag_13_Au_12_^2 +^, and exhibited a quantum fluorescence yield 200 times greater than the Au_25_ nanocluster, while product I did not.

**Figure 6:**
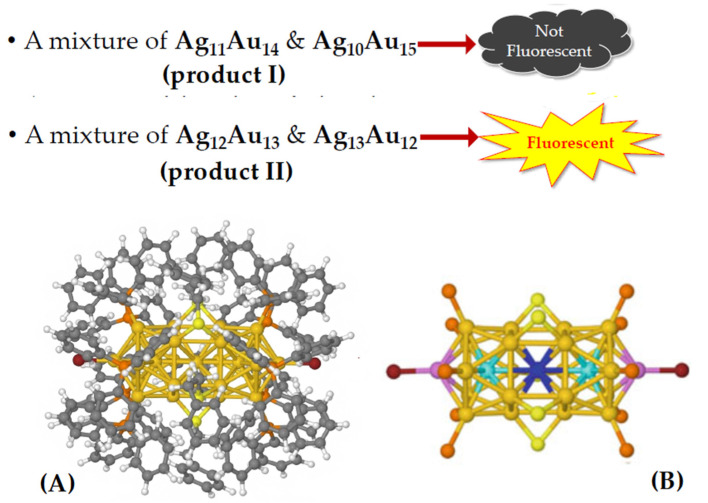
(**A**) Three-dimensional structure of products I and II; (**B**) schematic of the Ag_n_Au_25-n_^2+^ metal core: the ligands have been omitted for better clarity (save for P and S atoms), and the special central (*blue*), icosahedral central (*cyan*) and apical (*pink*) positions are marked by a special color.

The fluorescence performances of the two products are depicted in Figure 6, where the structure of the nanoparticles is also reported (A). In the same figure the schematic of the metal core is shown (B), without ligands (except S of the thiols and P of the phosphines), where the special central position, as well as the icosahedral central (*cyan*) and apical (*pink*) positions are colored in different colors.

**Figure 7:**
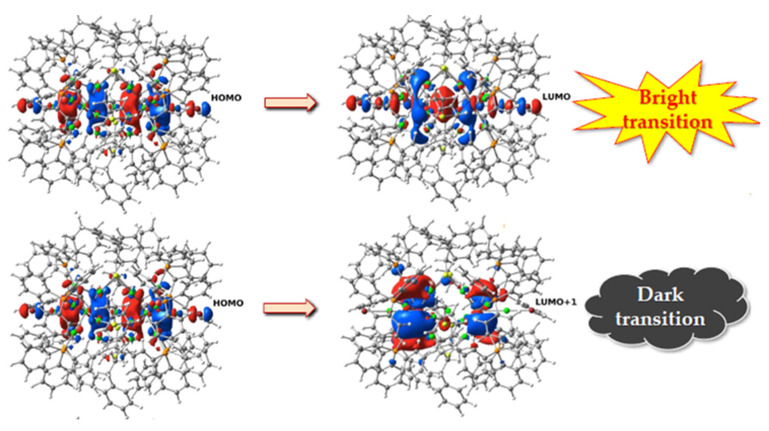
Isosurfaces of the occupied and virtual states in the electronic transition of the ligand-functionalized Ag_n_Au_25-n_^2+^ nanoalloys, when in the central position is present a silver atom (**upper**) or a gold atom (**lower**).

The TD-DFT calculations [30] show a complex scenario: we calculated many possible locations of Ag and Au in the 25 atomic positions for different stoichiometries. The electronic transitions S_1_ ← S_0_ are of the HOMO→LUMO type when the central position is occupied by a silver atom; when instead the central atom is Au, the electronic transitions S_1_ ← S_0_ have character HOMO→LUMO + 1 (see Figure 7). Furthermore, these transitions have significant oscillator strength (i.e., probability of occurring) only when they transitions are of the first type. Since the fluorescence emission is, in first approximation, due to a transition from the S_1_ state to S_0_, its probability depends mainly on the strength of the oscillator in the S_1_ ← S_0_ electronic absorption.

The increase in the quantum yield of fluorescence in product II is not due to the 13th Ag atom *per se*, nor to the possibility of placing more silver atoms than gold atoms: instead, (1) a higher silver content increases the possibility of having an Ag atom in the central position, and (2) above a certain threshold (incidentally, when the Ag content exceeds the Au content, i.e., n > 12 in Ag_n_Au_25-n_^2+^) this becomes particularly probable, since some peripheral positions are preferred by gold atoms.

This allows stabilizing the virtual state LUMO (mainly located on the central atom), allowing a significant emission of S_0_ ← S_1_ fluorescence.

**Figure 8:**
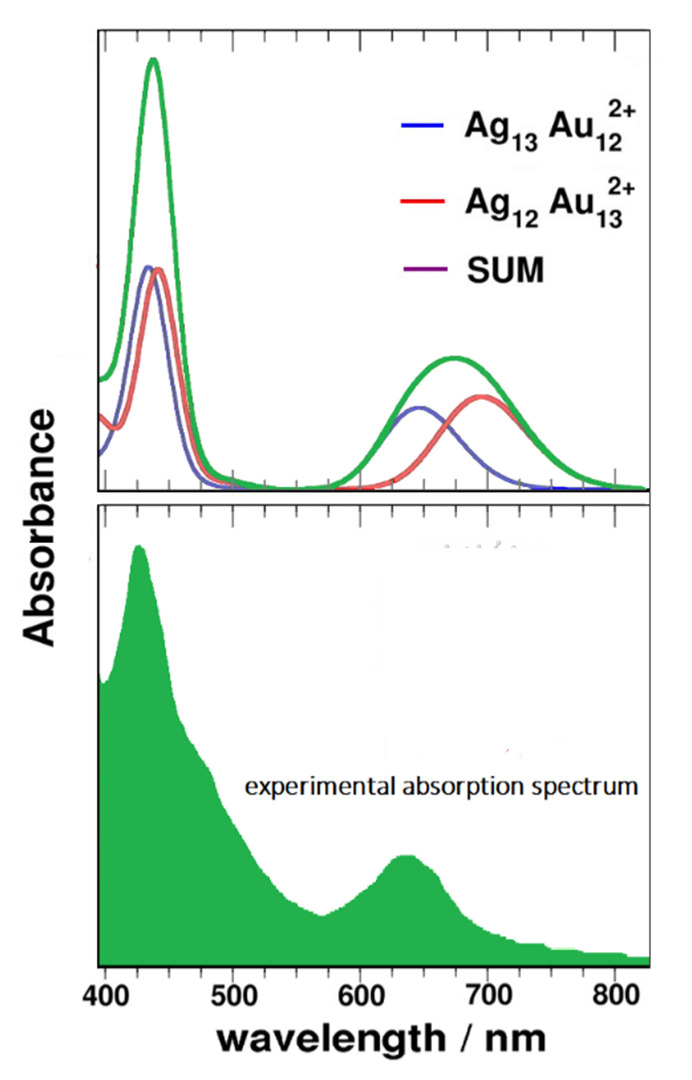
Simulated spectra of two Ag_n_Au_25-n_^2+^ (n = 12, 13) nanoalloys and their sum (upper); experimental absorption spectrum of product II (lower).

Furthermore, since product II is actually a mixture, its experimental absorption spectrum can be approximated by summing the absorption spectra calculated with n = 13, 12 (with central atom of silver), as shown in Figure 8. This computational prediction has been subsequent verified by ultrafast spectroscopic experiments [31], thus confirming the significance and efficacy of this TD-DFT approach.

Overall, this also proves that:

(1) Indeed a single swap in the Au/Ag relative composition can have dramatic effects on the optical properties, and in particular the fluorescence quantum yield, confirming the experimental finding;
(2) A quantum-chemistry based method such as linear approximation TD-DFT in combination with range-separated hybrid functionals can reproduce the effect of this small Au/Ag swap, a result that is far from trivial for particles whose core is made up of metal atoms.

At variance with this latter point, it has to be pinpointed however that including the effects of the environment on the DFT and TD-DFT calculation requires the use of codes that treat effectively periodic boundary conditions: with some exceptions, these tend to use plane waves as basis sets and, in turn, they privilege the use of nonhybrid exchange-correlation density functionals.

## 4. Recent Developments

Noble metal nanoclusters, protected by a monolayer of ligands are potential building blocks for advanced technologies in energy conversion, bioimaging, sensing and, mainly, heterogeneous catalysis, due to their quantum confinement effects. For these reasons, experimental and theoretical studies on such systems continue even in more recent years, with increasing intensity. Their electronic properties, which are intermediate between those of small molecules and plasmonic nanoparticles, can be exploited with quantum chemical investigations, primarily using DFT methods, which are able to predict observables such as densities of states, ionization energies, and optical spectra. In this last paragraph, I present a selection of the most recent and interesting works on these systems.

The research on atomically precise noble metal nanoclusters both from the synthetic point of view and from the theoretical study was illustrated by different review by C. M. Aikens [32] and Y. Du et al. [33]. In particular, thiolate-stabilized gold nanoclusters with different stoichiometries exhibited different luminescence properties: the symmetry of the systems and the rigidity of the cores induced different nuclear changes upon photoexcitation, with effect on the observed Stokes shifts.

In phosphine-stabilized nanoclusters with Au_11_ cores [34], DFT calculations showed that the gold cores had a large impact on the composition of HOMO and LUMO orbitals of the nanoclusters, leading to different optical absorptions.

Binding energies, HOMO−LUMO gaps, and absorption spectra were determined using DFT calculations in gold nanoclusters stabilized with thiolate or chloride [35], observing that absorption spectra appeared very similar regardless of the ligand used.

Electronic observables such as densities of states, ionization energies, and electronic absorption spectra were predicted for triphenylphosphine-protected gold nanoclusters [36] using DFT calculations and the superatomic model, where the 6s valence electron of Au(0) contributes to molecular orbitals delocalized across the metal core with shapes derived from a particle-in-a-sphere model.

Incorporation of different metals from gold in nanoclusters can enhance their properties as catalytic activity and luminescence [37,38,39]. Actually, the luminescence quantum yields of metal nanoclusters are generally low [15,40,41,42]. For this reason, numerous attempts of doping with heteroatoms were made also recently [43,44] in order to improve the light-emitting efficiency of monometallic nanoclusters, as well as their catalytic properties.

A series of Ag/Pt bimetallic clusters were computationally studied [45]. Lowest energy structures were determined by DFT calculations and time-dependent density functional theory was employed to calculate optical properties: in Ag/Pt nanoparticles with core−shell structure the optical properties were sensitive to both Pt concentration and cluster size.

Two hybrid gold−silver nanoclusters with dual-band emission [46] were theoretically studied using density DFT and time-dependent TD-DFT calculations. Hybrid functionals were found to successfully predict absorption and emission.

Thiolate-protected bimetallic nanoclusters [39] were analyzed in order to understand the relationship between electronic and catalytic properties. Actually, the study of the electronic properties of thiolate-protected bimetallic nanoclusters, especially the charge states and the HOMO−LUMO energy levels, is important because changes in these characteristics can be used to explain improved catalytic activities upon doping. In this case DFT calculations indicated that a lower Gibbs free energy led to the better catalytic performances of these nanoclusters when doped.

However, it is very complex to compare both experimental and computational results exposed in these papers, because the number and type of metal atoms vary, as well as the type of organic ligands, and the type of functionals adopted in the DFT calculations. In particular, as discussed in the previous section, effects due to environment are almost only taken into account using nonhybrid density functionals.

On the other hand, there is no unifying scheme or theoretical framework to fully interpret and predict the optoelectronic properties of noble metal nanoclusters, and a case by case approach has to be employed in order to elucidate these features.

## 5. Conclusions

The DFT method, with its time-dependent extension, is able to predict and describe the structural and spectroscopic properties of ligand-protected metal nanoparticles as shown by the investigations reported here and in the more recent bibliography referenced in the previous section. In particular, these calculations allow to adequately simulate the electronic absorption spectra and to clarify the role of ligands and the relationship between size, stoichiometry and optical properties of these nanoclusters. Sometimes, multiple models have to be built to correctly dissect and interpret the computed data, still they remain of paramount relevance to interpret data that otherwise would remain ambiguous (e.g., the origin of certain absorption/emission bands) or puzzling (e.g., the sudden increase of fluorescence swapping a single Au atom for a single Ag atom). All these computational studies are also important for the applications of functionalized noble metal nanoclusters in catalysis, optoelectronics and biomedicine, particularly as there is lack of a unifying scheme or theoretical framework to fully predict the optoelectronic features of this class of compounds. For instance, it is still not possible to reasonably foresee in which cases charge transfer metal → ligands occurs or instead the transitions are of the metal → metal type, without recurring to a case-by-case dissection of the electronic spectrum with ab initio methods.

Furthermore, an accurate dissection of the electronic spectra into atomic contributions is easier by (1) an approach based on hybrid or range-separated hybrid density functionals in conjunction with localized basis sets (benchmarked in detail in ref. [47]). However, the easiest way to include polarization effects due to the (crystal) environment is through (2) the use of nonhybrid functionals and delocalized plane wave-based basis sets, like it is done for instance in the group of Hannu Hakkinen (for a recent case see for example ref. [48]). The application of an approach based on method (1) in combination with polarized charges [49] is currently employed in a number of organic pigments assembled as molecular crystals [50,51,52,53,54], but it has not been yet adopted and adapted to the case of crystals including metal nanoclusters.

These limitations, if properly addressed, will lead to a breakthrough improvement in the description of the opto-electronic properties of metal-based nanoclusters that would pave the way to their much easier understanding and widespread use.
